# Factors influencing uptake of voluntary counselling and testing services for HIV/AIDS in the Lower Manya Krobo Municipality (LMKM) in the Eastern Region of Ghana: a cross-sectional household survey

**DOI:** 10.1186/s41043-015-0035-8

**Published:** 2015-11-21

**Authors:** Paschal A. Apanga, Robert Akparibo, John K. Awoonor-Williams

**Affiliations:** 1 Talensi District Hospital, Ghana Health Service, PMB, Tongo, Upper East Region Ghana; 2 School of Health and Related Research (ScHARR), University of Sheffield, Sheffield, UK; 3 Regional Health Directorate, Ghana Health Service, Bolgatanga, Upper East Region Ghana

**Keywords:** HIV, AIDS, VCT, Uptake, Awareness, Ghana

## Abstract

**Background:**

Voluntary counselling and testing (VCT) is one of the nine strategies recommended for prevention and control of HIV globally. In this study, we assessed the awareness and utilisation of VCT services among residents of the Lower Manya Krobo Municipality (LMKM) in the Eastern Region of Ghana.

**Methods:**

A population-based descriptive cross-sectional survey was conducted with 200 participants, aged between 18 and 55 years. Participants were recruited using cluster and simple random techniques to take part in the survey. Data was analysed descriptively, as well as using regression analysis approach.

**Results:**

Ninety-one percent of the respondents surveyed were aware of VCT services for HIV/AIDS. Seventy percent (70 %) have used VCT service in the last 12 months prior to the survey. Of this proportion, 97 % were satisfied with the quality of VCT services offered and indicated their willingness to recommend the service to others. Participants desire to know their HIV status (40 %), referral by health workers (25 %), and participants who wanted to get married (11 %) were the main reasons for increased uptake. Participants who had formal education, primary (OR = 1.8 (95 % CI 1.25–2.84)), junior high school (OR = 2.3 (95 % CI 1.54–3.37)), senior high school (OR = 2.8 (95 % CI 1.73–4.78)), and tertiary (OR = 3.4 (95 % CI 1.98–8.42)), had increased chance of using VCT service compared with participants who had no education (*p* < 0.001). Reasons for non-utilisation of VCT service were lack of awareness of the VCT service in the area (32 %), fear of being stigmatised (53 %), and the belief that HIV/AIDS cannot be cured and therefore the lack of need (5 %).

**Conclusions:**

Although awareness and utilisation of VCT service rates were reportedly high, more efforts need to be done in order to increase awareness and promote utilisation. HIV/AIDS educational campaign programmes need to be strongly pursued, with emphasis on the benefits of VCT services. This has the potential of reducing stigma and increase utilisation.

## Background

Sub-Saharan Africa has the most HIV cases in the world, accounting for 67 % of people living with HIV and 75 % of deaths due to HIV/AIDS [1]. Knowing one’s HIV status is an important step of the HIV prevention process [2]. In 2001, the UN General Assembly Declaration on commitment to fighting the HIV/AIDS menace made prevention of the disease a priority [3]. According to WHO, “everyone has a right to know their HIV status”, and in line with this, the adoption of voluntary counselling and testing (VCT) services has been recommended, which is one of the nine established prevention strategies, as an international HIV/AIDS screening and prevention policy [4]. Voluntary counselling and testing is a confidential discussion between a client and the health provider, after an informed consent has been obtained, to provide correct test results for the client [4]. The process consist of pre-test counselling, post-test counselling, and follow-up counselling, which are offered in designated VCT centres [4, 5]. Pre-test counselling involves counselling the client on the benefits of HIV testing before the client takes the HIV test, whilst post-test counselling involves counselling the client on evidence-based preventive measures and treatment of HIV/AIDS. Post-test counselling is often offered to both individuals who are tested negative or positive for HIV [4, 5]. Follow-up counselling on the other hand is offered to provide further information to the client to enforce pre and post-test counselling messages [5].

Globally, although the uptake of VCT services for HIV/AIDS has remained low, its significance is widely acknowledged [2]. It is an effective way of enhancing the knowledge of people about HIV and measures to take to prevent HIV/AIDS [2]. Particularly, people who test positive for HIV will know when to start treatment, as well as how to reduce the risk of co-infections and prevent AIDS mortality [4, 6, 7]. Furthermore, it is well documented that the risk of transmission of the HIV virus by an infected person to an uninfected person could be reduced by 96 % if HIV carriers are identified early through VCT and put on treatment [8]. Individuals who test negative will have the opportunity to protect themselves through evidence-based preventive measures such as safe sex, usage of condoms, and being faithful to one sexual partner [4, 8]. Most economic evaluation studies have further reported that VCT is cost-effective and can lead to a positive sexual behavioural change in high-prevalence settings and lowering of HIV/AIDS rates [9].

Evidence from South Africa and India has suggested that increased availability of VCT services for HIV, combined with media education, could increase the demand for VCT [10, 11]. However, in Ghana, very little data exists regarding the demand for VCT services, and in the Eastern Region, such information is virtually non-existence despite government effort to use VCT as a tool for controlling risks of infection. The Ghanaian government has consciously made the implementation of VCT for HIV approach, a priority since 2006 as a measure to reduce the risk of infection and transmission of HIV [12]. For instance, the Ghana Health Service (GHS), which is the government health implementing agency, in collaboration with its partners working in the health sector (including UNICEF, WHO, and USAID), has established VCT centres across the ten regions of the country since 2006. The agency has also identified and trained over 600 peer counsellors to provide counselling and testing services for HIV in these centres [13]. Although the VCT centres have been functional for over a decade now, no conscious and organised research has examined the awareness of community members, as well as utilisation of the service being offered. Only some recent GHS reports have mentioned that uptake of VCT services in many parts of the country has remained low, citing average utilisation rates of 7 % for women and 4 % for men [12, 14].

In the Lower Manya Krobo Municipality (LMKM) municipality, there are 15 functional VCT centres that were established between 2007 and 2012. Unfortunately, specific data regarding community members’ awareness and visitation of these centres to use the VCT services are unavailable. The municipality is one of the most populous in the Eastern Region [15], with a significant high rate of HIV infection above national average [12, 16]. Therefore, it is important to understand what needs to be done in order to increase demand for HIV counselling and testing and to reduce risk of infection and transmission. We conducted the survey to explore whether residents of the LMKM of the Eastern Region are aware of the existence of VCT services in the municipality, explore their utilisation of the service, as well as identify reasons for non-utilisation. We believe that through a small-scale cross-sectional study, findings of this study will be useful for planning effective strategies that can be implemented to increase the demand for VCT services for HIV in the municipality in particular and the Eastern Region in general.

## Methods

The survey was conducted between June and September 2013 in the LMKM, one of the 26 districts constituting the Eastern Region of Ghana. The LMKM has four hospitals, nine clinics, and two maternity homes [17]. Each of these facilities has a VCT centre attached to it that provides counselling and testing services for HIV to the residents [17]. We recruited 200 adult males and females aged between 18 and 55 years to take part in the survey, as this is the age group most likely to have increased risk of HIV infections in Ghana [18]. Furthermore, the selection of this group was also partly informed by the age range previous studies have used [19]. The 200 participants were selected using a convenient sampling method, which allowed us to select participants that were easily accessible within households [20–22]. Two participants each were selected from a random sample of 100 households, from 10 out of 56 villages that form the LMKM.

We estimated based on data available that if the awareness of VCT services in the municipality was around 15 %, then to estimate this proportion within ±5 %, that is, at 95 % confidence interval from 10 % to 20 %, required 196 responders [23, 24]. The 196 responders were approximated to the nearest tenth, which is equal to 200 responders. The 200 participants were then identified, approached, and recruited by the researchers themselves, with the support of a community volunteer recruited from each of the ten villages. With the help of the volunteer, the researchers first approached village heads/leaders from each to inform them about the study, as well as obtained permission from them to conduct the study: recruit and interview potential participants. Overall, a proper community entry protocol was observed, as this is the common practice for conducting research within the local community context in Ghana [25]. The community volunteers were highly respected members of their respective communities, who had good knowledge of the local residents, and we believe their presence in the team may have influenced the high response rate of our survey (100 %). All households were numbered, and a sampling interval starting point of *n* = 5 was used to select the first house. Subsequent selection of every 5th household then followed in the same direction.

All participants who agreed to take part were briefed about the study, and why they were being asked to participate. They were informed that participation was voluntary and that they have the right to withdraw even after they have been interviewed. The researchers then administered a questionnaire, designed with closed ended pre-coded questions, face-to-face to assess participants’ awareness of VCT services for HIV/AIDS in the community, their use of the service and barriers to, as well as facilitators of service utilisation. The questionnaire was first piloted on eight [8] participants in Somanya—a town in a neighbouring district which shares border with the LMKM. Results from the pre-test were used to validate the final questionnaire for the main survey. The responses gathered from the main survey were analysed using IBM SPSS version 21.0. All variables were changed from string to numeric variables, and were also recoded into new labels to conform to the SPSS statistical software. Descriptive analyses were used to estimate the proportion of respondents who were aware of VCT services, as well as ever used VCT services. Selected socio-demographic factors (age, gender, marital status, education level, and religion) were further analysed by using regression-modelling technique to assess whether these were potential barriers to uptake of VCT services.

Following the results of the descriptive analysis, it was more appropriate to collapse some of the variables such as marital status and religion before performing the logistic regression, as it did not make statistical sense to conduct such analysis because of the small sample sizes (Tables 1 and 5). Odd ratios, confident intervals, and *p* values were computed, with *p* < 0.05 depicting statistical significance. Ethical approval was obtained from both the Ghana Health Service and the University of Sheffield. The Lower Manya Krobo Municipal Health Directorate gave final permission for the study to be conducted in the municipality.

**Table 1:** Socio-demographic characteristics of 200 respondents who took part in the survey in Lower Manya Krobo Municipality of the Eastern Region

Demographic variable	Category of respondents	Frequency (*n*)	Percentage (%)
Gender	Male	107	54.0
	Female	93	46.0
Marital status	Married	98	49.0
	Single	98	49.0
	Divorced	2	1.0
	Widowed	2	1.0
Age group (years)	18–27	77	39.0
	28–37	52	26.0
	≥38	71	35.0
Religion	Christian	173	87.0
	Moslem	21	10.0
	Traditional	6	3.0
Education level	No education	31	15.0
	Primary	38	19.0
	JHS	52	26.0
	SHS	55	28.0
	Tertiary	24	12.0

Notes: results by description analyses

*SHS* senior high school, *JSH* junior high school

## Results

Table 1 summarises the results of the demographic characteristics of the 200 participants who took part in the survey. The mean age in the survey was 33.3 years, with the youngest age group (18–27 years) representing the majority (39 %) of the study participants. There were more male (54 %) compared to female (46 %) participants in the study. Participants who were classified as widows and divorced were least represented (Table 1). The majority of the respondents (87.0 %) were affiliated to the Christian religion, whilst the minority were followers of Islamic and African traditional religions (Table 1).

The main outcomes of interest were awareness of VCT services, uptake of VCT, and reasons for using or not using VCT services. To assess participants’ level of awareness and utilisation of services, we asked if participants were aware of any VCT services and/or have ever used the service being provided in the municipality in the last 12 months. Ninety-one percent of the respondents reported that they were aware of VCT services in the municipality. Seventy percent reported having visited VCT service centres at least once for voluntary counselling and testing for HIV in the last 12 months. Ninety-seven (97 %) indicated that they would recommend the service to any of their colleagues who are unaware of the existence of VCT services in the municipality. Table 2 summarises the participants’ responses regarding awareness, utilisation, and willingness to recommend service to other residents in the LMKM.

**Table 2:** Awareness, utilisation, and recommendation of VCT services (*n* = 200)

Variables	Responses	Frequency (*n*)	Percentage (%)
Aware of VCT services	Yes	181	91
Gone for VCT services	No	19	9
	Yes	140	70
	No	60	30
Will recommend VCT services to colleagues	Yes	136	97
	No	4	3

Note: results by description analyses

Results from the descriptive analysis also revealed the main reasons for residents’ use of VCT services for HIV/AIDS in the LMKM (Table 3). Of participants who ever used VCT service in the district, 40 % said they wanted to know their HIV status, 25 % said they used the service because they were referred by a health worker, 11 % indicated that it was a requirement by their church in order to get married to their partners (11 %), whilst 10 % undertook VCT because they wanted to plan a healthy sexual life. Among the non-service users, stigma (53 %), lack of knowledge/awareness of the service (32 %), and lack of need since HIV/AIDS cannot be cured (5 %) were the major reasons for non-utilisation (Table 4). Of those who indicated that they were using the service, nearly all (97 %) said they were willing to recommend the service to a colleague.

**Table 3:** Reasons for using VCT services (*n* = 140)

Response	Frequency (*n*)	Percentage (%)
I wanted to know my HIV status	56	40.0
To plan my sexual life	14	10.0
To seek early treatment if I am positive	4	3.0
I had unprotected sex	9	6.0
I was referred by a doctor/health worker	35	25.0
I wanted to get married	15	11.0
I have/had multiple sexual partners	7	5.0

Notes: results by description analyses

**Table 4:** Reasons for not using VCT services (*n* = 60)

Response	Frequency (*n*)	Percentage (%)
Stigmatisation of HIV/AIDS	32	53.0
Unaware of VCT services	19	32.0
No need, HIV/AIDS cannot be cured	3	5.0
I engage in only protected sex	2	3.0
I cannot contract HIV/AIDS	1	2.0
Test results are unreliable	1	2.0
Being positive will hasten my death	2	3.0

Notes: results by description analyses

The multivariate analysis exploring relationship between socio-demographic factors and utilisation of VCT services for HIV are presented in Table 5. As shown, the results suggest that there is a strong relationship between educational status and level of VCT service usage. Adjusted odd ratios suggest that participants who had up to primary level education, compared with no education, were 80 % more likely to use VCT service (OR = 1.8 (95 % CI 1.25–2.84)). The odd ratios for participants who were educated up to pre-tertiary or senior high school level (OR = 2.8 (95 % CI 1.73–4.78)) and tertiary level (OR = 3.4 (95 % CI 1.98–8.42)) show even high utilisation rate compared with participants with no education. Results on the other hand show that age, marital, religion, and gender status had no significant influence on VCT service use (Table 5).

**Table 5:** Logistic regression of demographic factors influencing VCT service utilisation

Variables	OR	95 % CI	*p*
Age (years)			
18–27	1.0		
28–37	0.8	(0.16–2.52)	(0.312)
≥ 38	1.3	(0.23–7.00)	(0.789)
Gender			
Male	1.0		
Female	1.1	(0.39–2.90)	(0.886)
Marital status			
Unmarried	1.0		
Married	1.1	(0.41–2.69)	0.909
Religion			
Non-Christians	1.0		
Christians	0.9	(0.18–2.38)	(0.529)
Education level			
No education	1.0		
Primary	1.8	(1.25–2.84)	(<0.001)
JHS	2.3	(1.54–3.37)	(<0.001)
SHS	2.8	(1.73–4.78)	(<0.001)
Tertiary	3.4	(1.98–8.42)	(<0.000)

Notes: results by multivariate analyses

*OR* odd ratios, *CI* confident interval, *p* probability value

## Discussion

This study adopted a population-based cross-sectional survey design to investigate awareness, utilisation, and reasons for use of VCT for HIV among residents of the LMKM of the Eastern Region of Ghana. The findings of the survey indicate that the majority of residents in the district generally are aware of the existence of VCT services for HIV, and their attitude towards the use of these services is favourable. As reported, awareness of VCT services among the study participants was high (90 %), with a corresponding significant high utilisation rate (70 %). These current findings are somewhat contrary to other previous studies that investigated VCT uptake in sub-Saharan Africa. For instance, Bwambale and colleagues investigated VCT service utilisation among men in rural Western Uganda [21], and reported a lower uptake rate (24.5 %) compared with what we found in the current study in Ghana (70 %). Studies on VCT service uptake [26] involving only tertiary education male students in Ghana also reported similar low uptake (20 %). Findings from previous studies are further supported by recent studies in Ethiopia where Leta and colleagues [7] examined uptake of VCT services among rural Ethiopian men. Overall VCT uptake rate in the Ethiopian study was 23.3 %, which is similar to the previous studies conducted in Uganda and Ghana. However, we can argue that these studies were conducted with only men, and findings might have been different if women were involved, as women are normally perceived to be more conscious about their health than their male counterparts. Furthermore, since some of the studies were conducted in early 2008, attitude towards utilisation of VCT services might have changed in recent times following the increased implementation of awareness-raising campaign programmes in most sub-Saharan African countries [27, 28].

In Ghana, efforts have been made by the government in collaboration with non-governmental organisations to increase communities’ awareness about HIV in particular, and the availability of VCT services in all districts, including LMKM. For instance, since 2007, the government in collaboration with development partners initiated HIV and VCT awareness campaign programmes in line with the Prevention of Mother to Child Transmission (PMTCT) of HIV strategy in all regions of the countries [29, 30]. These programmes might have contributed to the high awareness and utilisation rates reported in this survey. HIV and VCT awareness, as well as other health promotion campaigns in Ghana, has further been facilitated by the introduction of the Community-based Health Planning and Services (CHPS) policy. The CHPS policy was adopted in 1999 in order to increase rural access to healthcare while empowering local communities to take greater control over their health. Since then, several studies have reported on the impact of this strategy with increased awareness of health issues among the local community [31–34].

In Zambia and Kenya, the implementation of awareness-raising programmes has also led to increased awareness and uptake of VCT services. For instance, a recent study comparing awareness of VCT sites between residents of Lusaka (Zambia) and Kigali (Rwanda) has revealed that 79 % of residents in Lusaka compared to 56 % in Kigali was aware of voluntary counselling and testing sites in their communities, and was willing to use the service to know their HIV status (91 % Lusaka vs 47 % Kigali). Poor awareness rate in Kigali, Rwanda was attributed to lack of awareness programmes to educate residents on the existence of VCT services [28].

In this study, the participants who reported not using the service gave reasons such as not being aware of the service (32 %), stigma (53 %), and holding the perception that HIV is a non-curable disease and therefore the lack of need to know one’s status (Table 4). This thus suggests that the education messages may not be reaching everyone in the study setting or are not focused on reducing stigma related to HIV. Therefore, there is the need for the Ghana Health Service to re-visit the channels currently adopted to deliver VCT services awareness messages in rural communities of the Eastern Region. Harmonising and integrating these messages within existing health education programmes could help increase awareness, access, and utilisation. As reported in this survey, HIV-related stigma is still very high in the LMKM. Therefore, educational messages need to be more focused towards reducing stigma against people living with HIV/AIDS. This will help to reduce fear of stigma and increase interest and attitudes towards VCT services for HIV.

Individual behaviour and lifestyle factors such as desire to know one status, planning for a life partner, fear of contracting HIV because of unprotected sex, and having multiple sex partners were highly ranked as the main reasons for using VCT services. Age, marital status, religion, and gender did not influence VCT service utilisation. However, the findings suggest that the higher the educational level of respondents, the more likely that they will use VCT service. This is consistent with findings from several recent studies that also examined factors influencing VCT service utilisation in some sub-Saharan Africa and Southeast Asia [15, 21, 27, 28, 35]. Findings from these studies consistently highlight fear of stigma, level of education, and non-awareness as key reasons for non-utilisation of VCT service.

Our findings will be useful for policy and programme decision-making purposes in Eastern Region of Ghana, as there are currently no data regarding awareness and use of the Government-established VCT service centres across the country. However, some weaknesses in the study design limit the generalisation of the findings to the entire population of the study area. For instance, the selection of participants from households to participate in the study used a convenience rather than a random sampling approach, which may not be representative of the general population of residents in the study area. Although, we could argue that the households where the participants were recruited were selected using a random sampling method, and this might have contributed to minimise any problem of selection bias. Nonetheless, the generalisation of the study findings to the entire residents of the LMKM needs to be treated with caution. Also, the question on whether participants had previously tested for HIV was not asked, which is relevant and related to knowledge and attitude towards testing and utilisation of the service [28]. Finally, our study is limited because only a few socio-demographic variables were tested. It would have been useful also to assess whether socio-demographic factors such as income, occupation, and family size does affect VCT service uptake in the area. Our upcoming qualitative paper explores these issues in-depth.

## Conclusions

In conclusion, the survey has provided evidence to suggest that creating awareness among community members about VCT service for HIV could contribute to increased uptake of the service among local community members. On the other hand, lack of awareness can result in people not using such services even if such services were available and accessible. Furthermore, if people have the desire to know their HIV status, and if health workers make conscious effort to refer their clients to VCT service centres, this could contribute to increased uptake. For programming purposes, it is essential that more effort is made towards increasing awareness. HIV/AIDS education campaign programmes need to be seriously pursued, with emphasis on the benefits of VCT services. This will help reduce stigma and increase utilisation of VCT services in Ghana.
